# miRNA Expression in Anaplastic Thyroid Carcinomas

**DOI:** 10.1371/journal.pone.0103871

**Published:** 2014-08-25

**Authors:** Aline Hébrant, Sébastien Floor, Manuel Saiselet, Aline Antoniou, Alice Desbuleux, Bérengère Snyers, Caroline La, Nicolas de Saint Aubain, Emmanuelle Leteurtre, Guy Andry, Carine Maenhaut

**Affiliations:** 1 Institute of Interdisciplinary Research (IRIBHM), School of Medicine, Université libre de Bruxelles, Campus Erasme, Brussels, Belgium; 2 Institut Jules Bordet, Bruxelles, Belgium; 3 Université de Lille 2, Faculté de Médecine, Lille, France; 4 CHRU de Lille, Institut de Pathologie, Lille, France; 5 WELBIO, School of Medicine, Université libre de Bruxelles, Campus Erasme, Brussels, Belgium; University of Cordoba, Spain

## Abstract

Anaplastic thyroid carcinoma (ATC) is the most lethal form of thyroid neoplasia and represents an end stage of thyroid tumor progression. No effective treatment exists so far. In this study, we analyzed the miRNA expression profiles of 11 ATC by microarrays and their relationship with the mRNA expression profiles of the same 11 ATC samples. ATC show distinct miRNA expression profiles compared to other less aggressive thyroid tumor types. ATC show 18 commonly deregulated miRNA compared to normal thyroid tissue (17 downregulated and 1 upregulated miRNA). First, the analysis of a combined approach of the mRNA gene expression and of the bioinformatically predicted mRNA targets of the deregulated miRNA suggested a role for these regulations in the epithelial to mesenchymal transition (EMT) process in ATC. Second, the direct interaction between one of the upregulated mRNA target, the LOX gene which is an EMT key player, and a downregulated miRNA, the miR-29a, was experimentally validated by a luciferase assay in HEK cell. Third, we confirmed that the ATC tissue is composed of about 50% of tumor associated macrophages (TAM) and suggested, by taking into account our data and published data, their most likely direct or paracrine intercommunication between them and the thyroid tumor cells, amplifying the tumor aggressiveness. Finally, we demonstrated by *in situ* hybridization a specific thyrocyte localization of 3 of the deregulated miRNA: let-7g, miR-29a and miR-30e and we pointed out the importance of identifying the cell type localization before drawing any conclusion on the physiopathological role of a given gene.

## Introduction

Thyroid tumors are divided into encapsulated benign tumors (autonomous and follicular adenomas) and carcinomas. These carcinomas are themselves subdivided into differentiated carcinomas (follicular carcinomas (FTC) or papillary carcinomas (PTC)) which may evolve into the very aggressive and dedifferentiated anaplastic carcinomas (ATC) [1]. Despite its low frequency (<5% of all thyroid carcinomas), ATC contributes to 14–50% of the thyroid mortality cancer with a medium survival of 3 to 5 months [2], [3]. Benefits obtained from chemotherapy and radiation therapy remain only marginal and there is, until now, no alternative treatment [4], [5]. New therapeutic approaches are therefore needed. On the other hand, the ATC tissues contain tumor associated macrophages (TAM) which may represent up to 50% of the nucleated cells and show elongated thin ramified cytoplasmic extensions [6]. A relationship between the increased density of TAM type M2 and decreased survival was reported in thyroid cancer patients [6]–[9]. A direct correlation between the density of the presence of the TAM and the aggressiveness of the ATC has been suggested [6]–[9].

To identify the molecular mechanisms involved in tumor evolution, we have analyzed the mRNA expression profiles of 59 thyroid tumors (11 ATC and 48 PTC) using the Affymetrix microarray technology [10]. We have now integrated into these results the miRNA expression profiles of the same 11 ATC.

miRNA are non-coding RNA of approximately 22 nucleotides in length which can regulate the expression of more than several hundreds of genes following their binding to the 3′ untranslated region (UTR) of the mRNA target containing corresponding antisense sequences [11]. The ∼6 nt 5′ miRNA ‘seed’ region is the most crucial component for recognizing and binding to target sites [12]. miRNA regulate gene expression through translational inhibition (accounting for 16%) or mRNA degradation (accounting for 84%) [13]–[16]. Clearly, as a miRNA acts mostly through mRNA degradation [15], [16], we expect to see this reflected in the mRNA levels of its targets and thus in our microarray results.

As bioinformatics mRNA target prediction algorithms allow to predict for each deregulated miRNA a list of mRNA targets, we integrated the bioinformatically predicted mRNA target list to the deregulated mRNA list of the same 11 ATC obtained previously experimentally [10]. In this study, we have used the miRtarget2 algorithm [17] which is based on several criteria: the complementarity of the seed region, the observation that true sites tend to be conserved between species, the secondary structure (a target site is not likely to be functional if it is inaccessible to miRNA binding) as well as the location of the seed target in 3′UTR (a site in the middle of a long UTR is less likely to be functional).

The analysis of miRNA and mRNA gene expression of the same ATC samples and bioinformatic target prediction programs has highlighted a major deregulated transcription program that dictates ATC cells characteristics: the epithelial to mesenchymal transition (EMT). Moreover, a luciferase assay validated the direct interaction between the 3′UTR of LOX, a key gene in the EMT process, upregulated in the 11 ATC samples, and miR-29a, downregulated in the same 11 ATC.

Because the ATC tissue is composed of about 50% of TAM which are suggested to play a major role in the ATC aggressiveness, we have confirmed by *in situ* hybridization the thyrocyte derived tumor cell localization of 3 of the deregulated miRNA: let-7g, miR-29a and miR-30e.

## Materials and Methods

### Tissue samples

14 ATC tissues and 19 other thyroid tumors (5 PTC, 5 FTC, 4 FTA and 5 AA) were provided by different hospitals: Regional Reference Cancer Center of Lille (Lille, France), Pitié-Salpêtrière (Paris, France), Bordet Institute (Brussels, Belgium), Cancer Center of the Cliniques Universitaires Saint-Luc (UCL, Brussels, Belgium) and Catholic University of Leuven (KUL, Leuven, Belgium). All these tumors were compared to a common miRNA reference pool of 19 normal thyroid tissues which are adjacent to benign thyroid tumors. 11 of these 14 ATC samples were used for microarray studies and 3 of them were used for independent validation. Tissues were immediately dissected, placed on ice, snap-frozen in liquid nitrogen and stored at −80°C until processing. 4 of the studied ATC were also used for *in situ* hybridization and immunohistochemistry. Protocols have been approved by the ethics committees of ALL institutions. Written informed consent was obtained from all participants involved in the study.

### RNA purification

Total RNA was extracted from thyroid tissues using a Trizol reagent kit (Invitrogen), followed by purification on miRNeasy columns (Qiagen). The RNA concentration was spectrophotometrically quantified, and its integrity was verified by visualization using an automated electrophoresis system (Experion, Bio-Rad).

### Mutation screening

In order to determine the mutational status for TP53, BRAF, H-RAS, N-RAS, K-RAS, PIK3CA, β-catenin in the 11 ATC samples, the mRNA sequences containing the most frequent mutations were reverse-transcribed and amplified by PCR using appropriate primer pairs (primer sequences and PCR conditions described in [10]). The PCR products were sequenced by Big Dye Terminator cycle sequencing on an automated ABI Prism 3100 sequencer (Applied Biosystems, Foster City, USA).

### Microarray hybridization and data normalization

0.5–2 µg of total RNA from 11 ATC samples and from the pool of 19 normal thyroid tissues were labelled with Hyanin 3 or Hyanin 5 using the miRCURY LNA microRNA Power Labelling Kit (Exiqon), according to the manufacturer's protocol. The labelled RNA were purified on miRNeasy columns (Qiagen). Samples were hybridized using Corning Pronto! Microarray Hybridization Kit onto in-house-printed miRNA microarray slides (LNA microRNA ready-to-spot probe sets, V11.0, Exiqon), which contain 1269 miRNA (human-mouse-rat probes) spotted in duplicate. All hybridizations were performed with dyes swap. The microarray slides were washed under stringent conditions. A LOWESS normalization was carried out by using BRB-ArrayTools (Version 3.6.0).

### Search for deregulated mRNA or miRNA

For deregulated miRNA, we have selected miRNA which varied from the baseline by at least 1.5-fold in the 11 ATC samples (without any opposite regulation), called the miRNA ATC list (Table S4).

For deregulated mRNA in ATC previously hybridized by our group, we have selected mRNA which varied from the baseline by at least 2-fold in all the 11 ATC samples (without any opposite regulation), called the mRNA ATC list [10].

### Nonsupervised analyses

Nonsupervised analyses were performed on the basis of between-sample correlation distances. Multidimensional scaling (MDS, as implemented by R's isoMDS function) was performed on all human miRNA present on the microarray.

### Immunohistochemistry

Immunohistochemical analyses were performed on 6 µm thick sections prepared from 4 paraffin-embedded ATC tissues (4 of the 11 ATC studied by microarrays) and from 1 normal thyroid tissue, with the primary antibody directed against CD163 (Leica Biosystems, UK, Clone 10D6). The sections were deparaffinized, pretreated with CC1 (EDTA, pH 8.4) and incubated with the antibody at a 1∶100 dilution. The revelation was performed with a detection kit (Roche, ref. 760-501).

### Validation of miRNA expression data by quantitative real-time RT-PCR (qRT-PCR)

qRT-PCR was performed using TaqMan MicroRNA Assay kits according to the manufacturer's protocol (Applied Biosystems). miRNA specific cDNA were generated from 10 ng of total RNA using the TaqMan microRNA RT kit and the gene-specific RT primers from the TaqMan microRNA Assays (Applied Biosystems) according to the manufacturer's protocol. Relative quantification was calculated based on the Pfaffl Method [18]. Ct data were normalized to an internal control U6 snRNA.

All miRNA validations were done in triplicate on 6 ATC (3 of the 11 ATC studied by microarrays and 3 independent ones).

### In situ hybridization of miRNA

Six µm thick paraffin sections of 4 ATC tissues (4 of the 11 ATC studied by microarrays) and of 1 normal thyroid tissue were mounted on Super frost+glass slides and deparaffinized. The slides were hybridized with 40 nM of the LNA miRNA let-7g, miR-29a and miR-30e probes (Exiqon) and the nuclei were counterstained with the nuclear fast red counterstain (Vector Laboratories, Burlingname, CA) by Bioneer as described in [19]. A scramble miRNA and miR-126 were used as negative and positive controls respectively. LNA-miRNA sequences are described in Table S1.

### Search for bioinformatically predicted mRNA targets of miRNA

A list of 79 mRNA targets of the 18 deregulated miRNA in ATC was constructed. This list resulted from the intersection of the bioinformatically predicted mRNA targets of the 18 deregulated miRNA (using miRTarget2 software with a miRTarget2 score >80% [17]) and the mRNA ATC list (taking into account the direction of the regulation). For instance, predicted mRNA targets of downregulated miRNA should be upregulated in our samples and thus were intersected with the list of mRNA upregulated in ATC (as described above). One of them, the LOX gene, predicted to be targeted by the downregulated miRNA-29a, was selected for the luciferase assay.

### Luciferase assay

A PCR fragment of ≈400 nucleotides encompassing the miR-29a binding site at the end of the 3′UTR of LOX (nucleotides 2941 to 3357) was cloned downstream of the Renilla luciferase gene, in the psiCHECK2 vector (Promega) (primers used for plasmid construction are listed in Table S2). The HEK293 cell line (derived from human embryonic kidney) was used for transfection experiments. The cells were seeded on 24-well plates in a DMEM (Dulbeccos's modified eagle medium, GIBCO), L-glutamine and sodium pyruvate medium until 50% of confluence prior to transfection. 100 ng of the luciferase vector was co-transfected with 50 nM of mimic miR-29a (Dharmacon) or a scramble and with 2 µl of LipofectAMINE 2000 (Invitrogen), according to the manufacturer's instructions. 24 h and 48 h after the transfection, luciferase activity was measured using the Dual Luciferase Reporter Assay system (Promega) using a MicroaLumatPlus (EG&G Berthold) and was normalized by measuring the firefly luciferase activity. Results were expressed as ratios of the normalized luminescence value of the cells co-transfected with the luciferase vector with the miR binding site wild-type or mutated in the seed sequence and the mimic miRNA compared to the normalized luminescence value of the cells co-transfected with the luciferase vector with the miR binding site wild-type or mutated in the seed sequence and the scramble. Primers used for directed mutagenesis are provided in Table S2.

### Statistical analyses

#### TAM quantification

After Immunohistochemistry detection with the primary antibody directed against CD163, 500 nucleated cells were counted on four different ATC tissues samples and on one normal adjacent tissue and the marked cells were counted for TAM quantification. Mean and standard deviation were then calculated.

#### Luciferase assay

The luciferase assay was performed in triplicates in 3 to 4 independent cell cultures for each of the 3 conditions (the luciferase vector with the miR binding site wild-type after 24 h or 48 h of transfection or the luciferase vector mutated in the seed sequence after 48 h of transfection). A t-test was performed on the ratios of normalized luminescence values (as described in the Luciferase assay section).

## Results

### Mutational screening of p53, BRAF, PIK3CA, H-RAS, K-RAS, N-RAS and β-catenin in the 11 ATC

We searched for the presence of the most common mutations reported for ATC. On the 11 ATC, p53 mutation was found in 4 (36.4%), BRAF mutation in 2 (18%), PIK3CA mutation in 1 (10%). One sample showed both BRAF and p53 mutations (ATC1). No mutation was found for RAS (H-RAS, K-RAS and N-RAS) or for β-catenin.

### The 11 ATC showed distinct miRNA expression profiles from the other thyroid tumors

**Figure 1:**
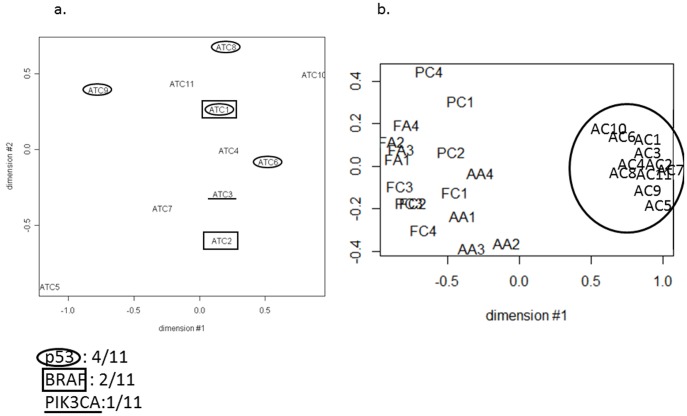
Multidimensional scaling (MDS) of the miRNA expression data: a. from 11 ATC (the ATC are labeled according to their mutation as depicted in the figure), b. from 11 ATC and 5 PTC (PC), 5 AA (AA), 5 FTC (FC) and 4 FTA (FA). All human miRNA present on the arrays were considered for the analyses.

The molecular phenotypes of ATC can best be demonstrated by a comprehensive microarray analysis of mRNA and miRNA expressions in these tissues. mRNA expression profiles of 11 ATC were obtained previously [10], and we have now performed miRNA expression analysis on the same tumors samples. Because of the absence of normal tissue counterparts for ATC, all gene expression profiles were compared to a common miRNA reference pool of 19 normal tissues which are adjacent to benign thyroid tumors. Overall miRNA expressions profiles from the 11 ATC, as well as from 5 PTC, 5 AA, 5 FTC and 4 FTA for comparison, were analyzed using a multidimensional scaling (MDS) (Figure 1). The MDS algorithm reduces the n-dimensions space (n: number of probes) into two dimensions while preserving the distances between the samples, and thereby visualizes the similarity relationships between them. MDS showed, firstly, that the ATC did not cluster according to their mutational status (Figure 1a) and secondly, that ATC miRNA expression profiles are clearly distinct from those of the other thyroid tumor types (Figure 1b).

### Deregulated microRNA in ATC

ATC presented 18 commonly deregulated miRNA (Table S4), called the miRNA list, with 17 downregulated and 1 upregulated miRNA (selection criteria described in Material and Methods). These 18 miRNA were significantly deregulated only in the ATC samples, and not in the other tumor types, except for miR-144 which was downregulated in all the thyroid tumor types (Table S4).

### About 60% of the nucleated cells in the ATC tissues are TAM

**Figure 2:**
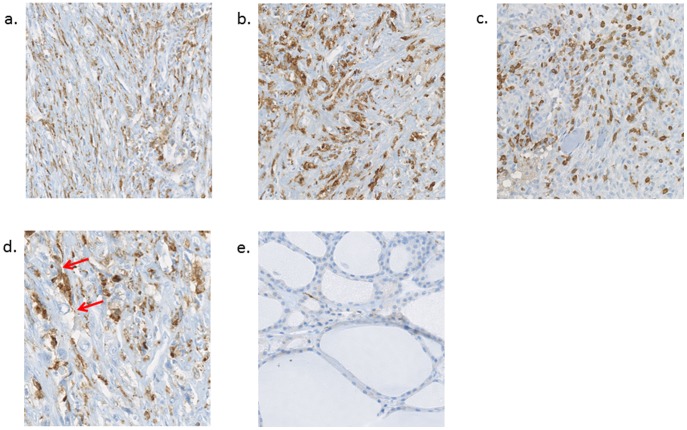
Immunohistochemistry with the CD163 antibody on 3 different ATC and on normal thyroid tissue. a, b, c: 20× magnification showing that about 50% of the nucleated cells are TAM, d: 40× magnification showing elongated cytoplasmic extensions in TAM as illustrated by the red arrows; e: 20× magnification illustrating the almost absence of TAM in normal thyroid tissue.

Because TAM were shown to be present in ATC and to have a major role in the aggressiveness of the tumors [6], we have investigated their proportion by IHC with the macrophage-specific CD163 antibody in 4 ATC samples studied for their miRNA profiles and a normal thyroid tissue as control. ATC tissue samples show 63%+/−4,6% (mean+/− standard deviation) of TAM among the nucleated cells (IHC are presented in Figure 2.a,b,c) which are almost not present in the normal thyroid tissue (Figure 2.e) (3%+/−1% (mean +/− standard deviation) of TAM among the nucleated cells). Moreover TAM present thin and elongated cytoplasmic extensions forming a canopy structure all over the tumor tissue (as illustrated in Figure 2.d).

### Validation of miRNA expression data

#### By literature

50% of our deregulated miRNA are common with the miRNA modulated in the three ATC cases studied by Braun et al. [20]: let-7f, let7-g, miR-29a, miR-30a, miR-30e, miR-125b, miR-141, miR-148b and miR-200b. MiR-30a and miR-125b were also previously reported as deregulated in another study [21], [22] (Figure S1).

#### By quantitative real-time RT-PCR (qRT-PCR)

To further validate the regulations observed by microarray, qRT-PCR was performed with miR-514, miR-144, miR-125b, miR-30a, let-7g, miR-29a, miR-34b, and miR-659, on 6 ATC. Three of them were part of the microarray tumor set, and the other three were new and independent ones.

The modulations of all the miRNA were confirmed except for miR-34b (Figure S1).

#### By in situ hybridization

Because ATC tissues are composed of about 50% of TAM which are suggested to play a major role in the ATC aggressiveness, we have performed *in situ* hybridization on four ATC (which were a part of the microarray tumor set) for 3 of the deregulated miRNA in order to localize the miRNA (e.g. thyrocyte derived tumor cell or TAM).

**Figure 3:**
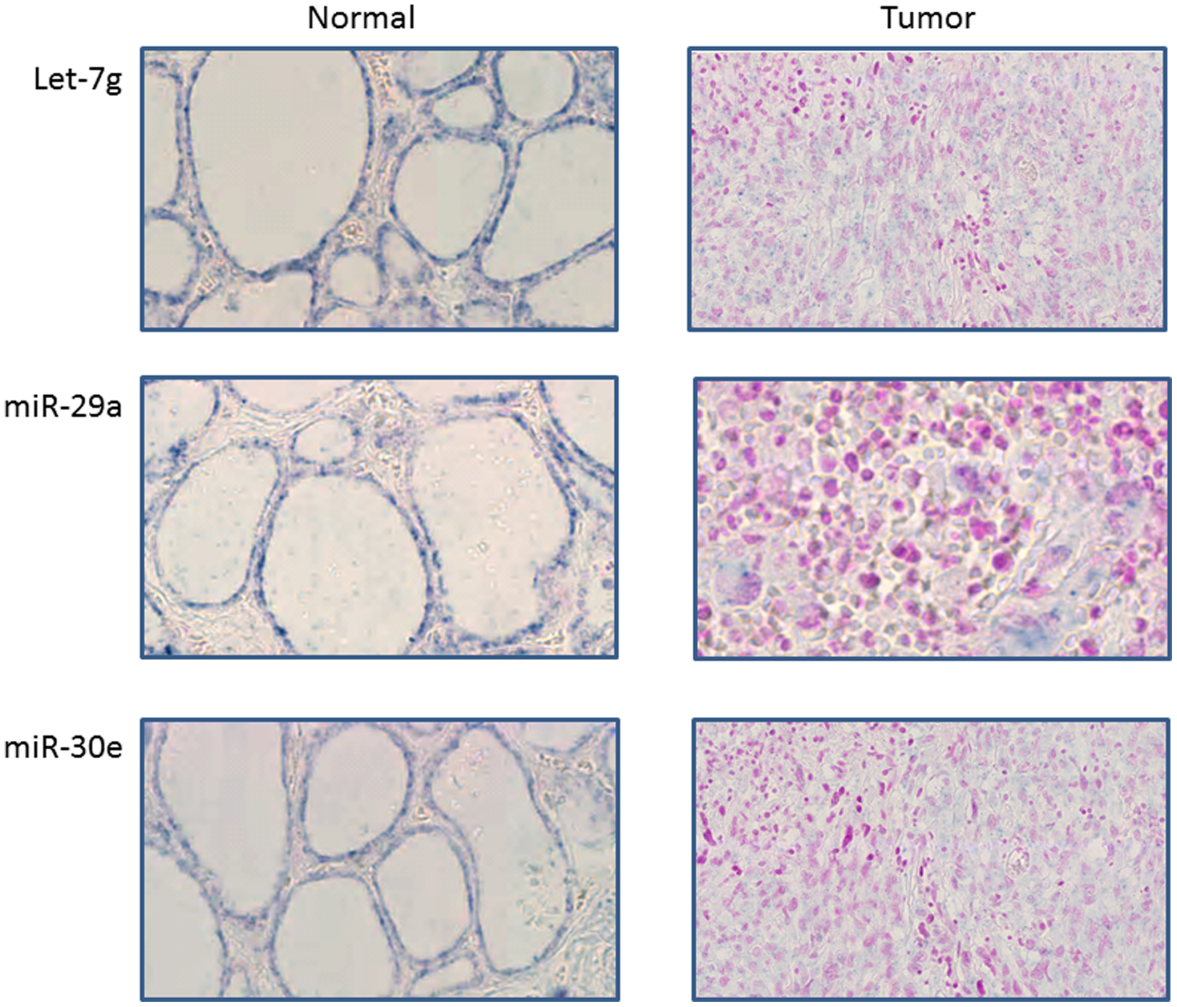
*In situ* hybridization of Let-7g, miR-29a and miR-30e in normal and ATC tissues. Tissue sections from normal and tumor thyroids were incubated with a full length DIG-labeled LNA probe to detect Let-7g, miR-29a and miR-30e: 20× magnification showing a strong miRNA signal observed (in blue) in the normal thyrocytes and a weak signal observed in the tumor cells derived from thyrocytes. No signal was detected in the TAM. 4 ATC were investigated. Representative pictures of 3 of them are shown.

The normal tissue section shows a strong ISH staining signal obtained with the LNA probes for Let-7g, miR-29a and miR-30e, which are located in thyrocytes. On the contrary, a very weak ISH signal for these 3 miRNA was observed in the four ATC samples. These miRNA were confirmed by a pathologist to be in tumor cells originated from thyrocytes and not in TAM (Figure 3). The 4 ATC investigated showed strong staining intensities with the miR-126 positive control probe and almost no signal for the scramble probe (data not shown).

### Analysis of the putative mRNA targets of deregulated miRNA in the same 11 ATC samples by using DAVID software

The biological action of miRNA is carried out by silencing the expression of their target genes. In order to highlight the role of the 18 miRNA which are deregulated in the ATC, a list of 79 mRNA targets (Table S3) of the 18 deregulated miRNA (obtained as described in Material and Methods) was analyzed using DAVID software – KEGG pathway. The only significantly altered function (FDR score <0.05) highlighted by this analysis is called ‘cytoskeleton’ and is related to the EMT process. This suggests that EMT, a major characteristic of ATC already observed in the mRNA expression analysis [10], might be directed by the deregulation of miRNA in ATC, as already suggested previously [20].

### Luciferase assay validates the direct interaction of LOX mRNA with miR-29a

**Figure 4:**
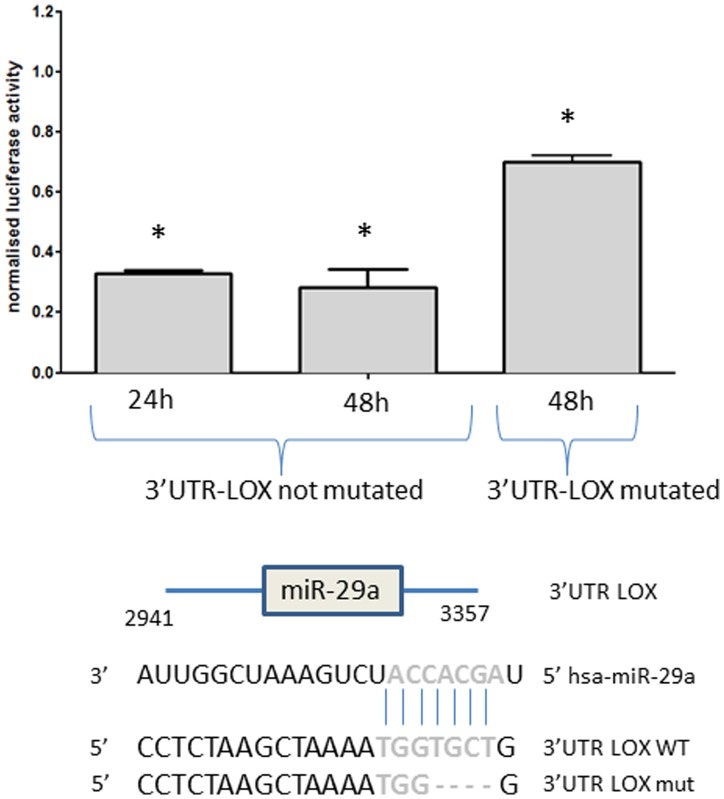
Normalized luciferase activity in HEK293 cells cotransfected during 24 h or 48 h with the 3′UTR-LOX construction or with the mutated construction (3′UTR-LOX mutated: partial deletion of the seed sequence) and with miR-29a or with the scramble. The seed sequence is represented in blue and the * indicates a p-value<5%.

Among the mRNA targets, the LOX gene predicted to be targeted by the downregulated miR-29a, was selected for a luciferase assay in order to validate their direct interaction which has not been previously described. The LOX gene is a key player in EMT and was shown to be an interesting therapeutic target [23]; it is upregulated at mRNA and protein level in our ATC samples (data not shown). About 400 nucleotides of the 3′UTR of LOX containing the seed sequence of the miR-29a were cloned in the psiCHECK2 vector and HEK293 cells were co-transfected with the vector and with the mimic miR-29a or with a mimic scramble as control. Luciferase activity, measured after 24 h and 48 h, was significantly decreased in the miR-29a transfected cells compared to the scramble transfected cells (p-value>5%). Deletion of a part of the seed sequence in the vector restored partially the luciferase activity showing the specificity of the binding of the miR-29a on this part of the 3′UTR of LOX (Figure 4). These results suggest that the miR-29a downregulates LOX mRNA through a direct interaction with its 3′UTR.

## Discussion

Many studies support the idea that miRNA play a role in tumorigenesis. This study was undertaken to further characterize the molecular mechanisms underlying ATC pathogenesis. To achieve this goal, the same 11 ATC samples previously analyzed for mRNA expression [10] have now been investigated for miRNA expression allowing a direct correlation between both expression profiles, as each miRNA can regulate many mRNA. MiRNA are often considered as fine-tunes regulators of the genome.

miRNA expression analysis was performed on 11 ATC and on 19 other less aggressive thyroid tumors for comparison including PTC, FTC, FA and AA. 17 downregulated miRNA and only one upregulated miRNA were identified as commonly deregulated in all ATC compared to a normal thyroid pool. This general downregulation of miRNA is also observed in many other cancers [24]. While the patterns of mRNA expressions in PTC and ATC are largely overlapping and show a distinct, but mostly quantitative evolution from one to the other [10], the overall downregulation of miRNA in ATC and their specific miRNA expression not found in the other tumor types can better explain the dramatic qualitative switches in pathology and clinical evolution. Another hypothesis explaining this physiopathology switch might be the high presence of TAM observed by others [6] and confirmed by immunohistochemistry in our ATC tissues (about 60% of the nucleated cells), while the other tumor types are less infiltrated by TAM [6]. TAM show thin, elongated cytoplasmic extensions that form a canopy structure all over tumor tissue. Paracrine or direct communication via the cytoplasmic extensions between the TAM and the thyroid tumor cells are often mentioned and are suggested to enhance tumor progression [25]–[27]. *In situ* hybridization experiments have assessed that the downregulated let-7g, miR-29a and miR-30e are present in tumor cells derived from thyrocytes and not in TAM. In the case of very heterogeneous tumors, such as ATC, it is thus essential to carefully verify the exact cellular localization of any transcript or protein identified by whole tissues analyses, before drawing any conclusion on its physiological role.

**Figure 5:**
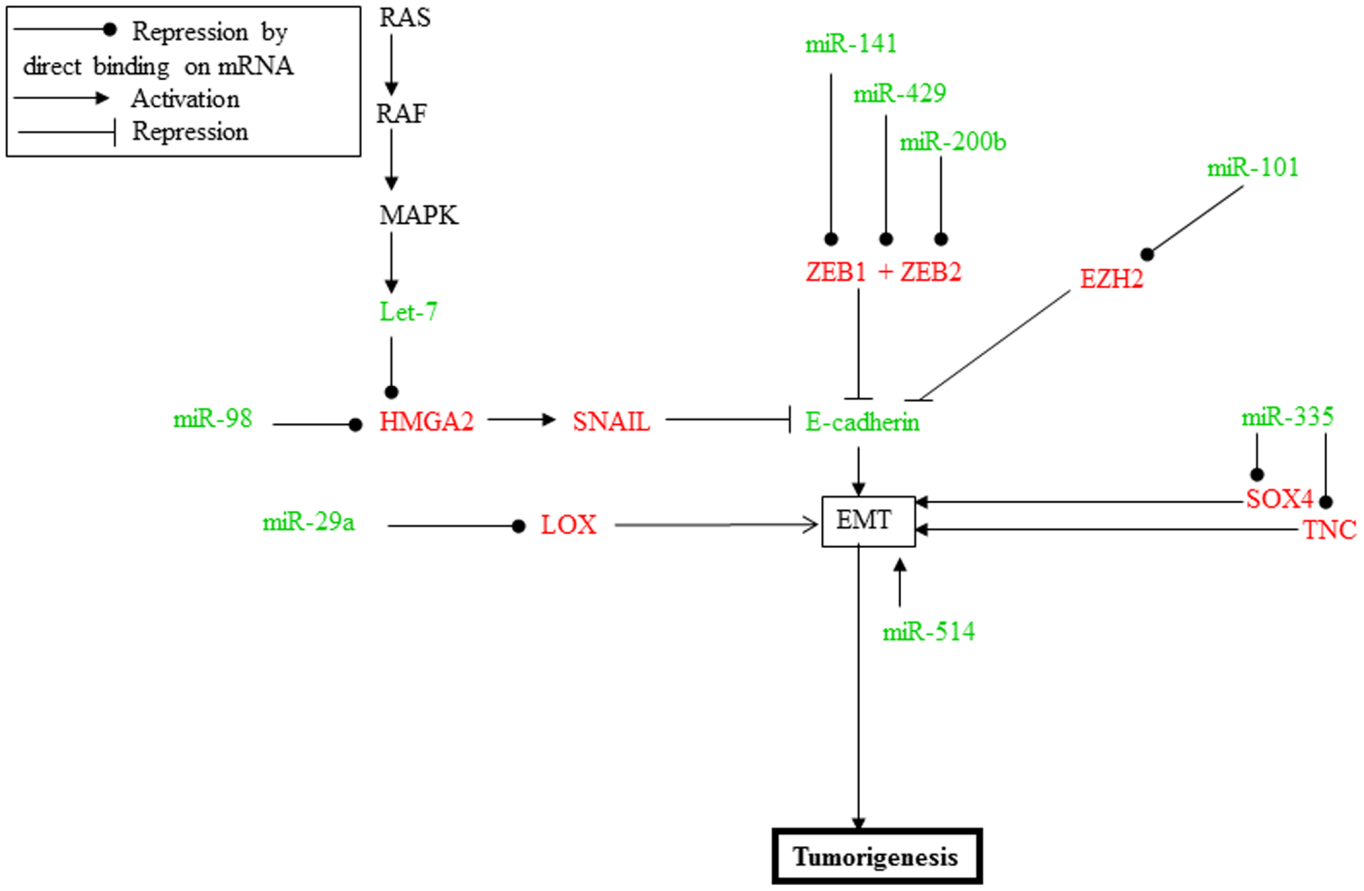
Proposed scheme synthetizing the miRNA and mRNA expression analyses of the same 11 ATC and highlighting the EMT feature of this tumor. Only direct binding of miRNA on mRNA are represented (green: downregulated m(i)RNA; red: upregulated m(i)RNA.

Studying the function of the mRNA targets of the deregulated miRNA should highlight a role of these miRNA in the pathogenesis of the ATC. The KEGG analysis pointed out the EMT process. Indeed, ATC are highly invasive, and part of patients die because of overwhelming distant metastases disease involving an EMT process. Moreover, the ATC tissue is completely dedifferentiated, without any structure anymore, and the cells present a mesenchymal morphology. The EMT process is the consequence of a molecular reprogramming of the cells consisting in an increased expression of mesenchymal type genes, like the mesenchymal N-cadherin (CDH2) which facilitates tumor cell binding to the stroma, and in a decreased expression of epithelial type genes, like E-cadherin (CDH1) which allows cell-cell interactions. TAM can induce the EMT process for example through paracrine TGF-β signalling and consecutive activation of the β-catenin pathway [26] or through TLR4/IL10 signaling [25]. The EMT switch may also be the consequence of the action of various miRNA modulating the expression of different mRNA, as synthetized in Figure 5.

In the following paragraphs, miRNA regulations which are not significant in all the ATC are specified by the fraction of positive tumors (no specification for those significantly alterated in all our 11 ATC). In our mRNA data [10], CDH1 is downregulated and CDH2 is upegulated in ATC [10]. CDH1 is transcriptionally repressed by SNAIL, ZEB1, ZEB2 and TWIST, and induces subsequent breakdown of cell junction and the ensuing loss of cell polarity [28]. SNAIL, ZEB1, ZEB2 and TWIST are all upregulated in ATC samples [10]. Three members of the miR-200 family, miR-141, miR-200b and miR-429, downregulated in our ATC (11/11, 11/11 and 9/11 respectively), have been shown to silence the ZEB proteins [29]–[32]. In addition, miR-101 (downregulated in 10/11 ATC) targets EZH2 (upregulated in all the ATC [33]), and mediates transcriptional silencing of CDH1 [34].

Activation of the RAS/MAPK pathway also induces EMT, together with HMGA2 upregulation. Let-7, for which many members of this family of miR are downregulated in our ATC samples, inversely regulates HMGA2 [35], upregulated in our data at mRNA and protein levels (data not shown). HMGA2 itself induces the expression of SNAIL, a transcriptional repressor of E-cadherin [35], [36]. This process can be initiated, for example, via the oncostatin M which can be secreted by M2 type macrophages and which downregulates miR-200 and let-7 family members and finally induces the EMT process [36]. Also, mir-98, downregulated in all our ATC tumors, negatively regulates HMGA2. The reintroduction of miR-98 accelerated the inhibition of glioma cell invasion and can therefore be interesting for further characterization for ATC therapy [37]. Another miRNA promoting the EMT process is miR-335 (downregulated in 10/11 ATC) which targets SOX4 and TNC (upregulated in 10/11 and 11/11 ATC respectively) [38], [39]. Moreover, we have now validated the direct interaction between the downregulated miR-29a and the upregulated LOX mRNA, which is also an EMT key player [23], suggesting another way for cancer cells to promote EMT.

Benefits obtained from chemotherapy and radiation therapy of ATC remain only marginal and there are no alternative treatments yet [4], [5], therefore new therapeutic approaches are needed. Our results suggest several approaches: first, detection of a circulating miRNA in the plasma, present in a remarkably stable form because protected from endogenous RNase activity, may allow to predict diagnosis or prognosis and eventually to treat the patients earlier [40]. This could be tested for the upregulated miR-659. Second, reinduction of one or more miRNA, downregulated in tumor cells, may be an interesting approach to treat the ATC patients but, of course, it raises the question of cell specificity. Third, as our results highlight a high proportion of TAM in ATC, and as TAM is pro-tumorigenic in advanced thyroid cancer, they could be then targeted pharmacologically [41].

## Supporting Information

Figure S1 Expression levels of 13 miRNA measured by qRT-PCR in 3 ATC studied by microarrays and in 3 ATC independant or obtained from the literature. The microarray expressions are included for comparision. Log_2_ ratios represent the expression ratios of the genes in the tumors versus the normal tissues.
(PPT)

Table S1 (XLSX)

Table S2 (XLS)

Table S3 (XLSX)

Table S4 **Deregulated miRNA in the 11 ATC samples and their corresponding values in papillary carcinoma (PC), follicular carcinoma (FC), follicular adenoma (FA) and autonomous adenoma (AA).** MiRNA expression values are in bold if they varied from the baseline by at least 1.5-fold (log_2_ of expression ratios).
(PPTX)

## References

[1] Smallridge RC, Marlow LA, Copland JA (2009) Anaplastic thyroid cancer: molecular pathogenesis and emerging therapies. Endocr Relat Cancer 16: 17–44. 18987168 10.1677/ERC-08-0154 PMC2829440

[2] Ain KB (1999) Anaplastic thyroid carcinoma: a therapeutic challenge. Semin Surg Oncol 16: 64–69. 9890741 10.1002/(sici)1098-2388(199901/02)16:1<64::aid-ssu10>3.0.co;2-u

[3] Nagaiah G, Hossain A, Mooney CJ, Parmentier J, Remick SC (2011) Anaplastic thyroid cancer: a review of epidemiology, pathogenesis, and treatment. J Oncol 2011: 542358. 21772843 10.1155/2011/542358 PMC3136148

[4] Are C, Shaha AR (2006) Anaplastic thyroid carcinoma: biology, pathogenesis, prognostic factors, and treatment approaches. Ann Surg Oncol 13: 453–464. 16474910 10.1245/ASO.2006.05.042

[5] Cooper DS, Doherty GM, Haugen BR, Kloos RT, Lee SL, et al (2006) Management guidelines for patients with thyroid nodules and differentiated thyroid cancer. Thyroid 16: 109–142. 16420177 10.1089/thy.2006.16.109

[6] Caillou B, Talbot M, Weyemi U, Pioche-Durieu C, Al GA, et al (2011) Tumor-associated macrophages (TAMs) form an interconnected cellular supportive network in anaplastic thyroid carcinoma. PLoS One 6: e22567 10.1371/journal.pone.0022567 [doi];PONE-D-10-05881 [pii] 21811634 PMC3141071

[7] Kim S, Cho SW, Min HS, Kim KM, Yeom GJ, et al (2013) The expression of tumor-associated macrophages in papillary thyroid carcinoma. Endocrinol Metab (Seoul) 28: 192–198 10.3803/EnM.2013.28.3.192 [doi] 24396678 PMC3811699

[8] Ryder M, Ghossein RA, Ricarte-Filho JC, Knauf JA, Fagin JA (2008) Increased density of tumor-associated macrophages is associated with decreased survival in advanced thyroid cancer. Endocr Relat Cancer 15: 1069–1074 ERC-08-0036 [pii]; 10.1677/ERC-08-0036 [doi] 18719091 PMC2648614

[9] Qing W, Fang WY, Ye L, Shen LY, Zhang XF, et al (2012) Density of tumor-associated macrophages correlates with lymph node metastasis in papillary thyroid carcinoma. Thyroid 22: 905–910 10.1089/thy.2011.0452 [doi] 22870901 PMC3429273

[10] Hebrant A, Dom G, Dewaele M, Andry G, Tresallet C, et al (2012) mRNA expression in papillary and anaplastic thyroid carcinoma: molecular anatomy of a killing switch. PLoS One 7: e37807. 23115614 10.1371/journal.pone.0037807 PMC3480355

[11] He L, Hannon GJ (2004) MicroRNAs: small RNAs with a big role in gene regulation. Nat Rev Genet 5: 522–531 10.1038/nrg1379 [doi];nrg1379 [pii] 15211354

[12] Kiriakidou M, Nelson PT, Kouranov A, Fitziev P, Bouyioukos C, et al (2004) A combined computational-experimental approach predicts human microRNA targets. Genes Dev 18: 1165–1178. 15131085 10.1101/gad.1184704 PMC415641

[13] Bagga S, Bracht J, Hunter S, Massirer K, Holtz J, et al (2005) Regulation by let-7 and lin-4 miRNAs results in target mRNA degradation. Cell 122: 553–563. 16122423 10.1016/j.cell.2005.07.031

[14] Du T, Zamore PD (2005) microPrimer: the biogenesis and function of microRNA. Development 132: 4645–4652. 16224044 10.1242/dev.02070

[15] Djuranovic S, Nahvi A, Green R (2011) A parsimonious model for gene regulation by miRNAs. Science 331: 550–553. 21292970 10.1126/science.1191138 PMC3955125

[16] Guo H, Ingolia NT, Weissman JS, Bartel DP (2010) Mammalian microRNAs predominantly act to decrease target mRNA levels. Nature 466: 835–840. 20703300 10.1038/nature09267 PMC2990499

[17] Wang X, El Naqa IM (2008) Prediction of both conserved and nonconserved microRNA targets in animals. Bioinformatics 24: 325–332. 18048393 10.1093/bioinformatics/btm595

[18] Pfaffl MW (2001) A new mathematical model for relative quantification in real-time RT-PCR. Nucleic Acids Res 29: e45. 11328886 10.1093/nar/29.9.e45 PMC55695

[19] Nielsen BS, Jorgensen S, Fog JU, Sokilde R, Christensen IJ, et al (2011) High levels of microRNA-21 in the stroma of colorectal cancers predict short disease-free survival in stage II colon cancer patients. Clin Exp Metastasis 28: 27–38 10.1007/s10585-010-9355-7 [doi] 21069438 PMC2998639

[20] Braun J, Hoang-Vu C, Dralle H, Huttelmaier S (2010) Downregulation of microRNAs directs the EMT and invasive potential of anaplastic thyroid carcinomas. Oncogene 29: 4237–4244. 20498632 10.1038/onc.2010.169

[21] Visone R, Pallante P, Vecchione A, Cirombella R, Ferracin M, et al (2007) Specific microRNAs are downregulated in human thyroid anaplastic carcinomas. Oncogene 26: 7590–7595. 17563749 10.1038/sj.onc.1210564

[22] Pallante P, Battista S, Pierantoni GM, Fusco A (2014) Deregulation of microRNA expression in thyroid neoplasias. Nat Rev Endocrinol 10: 88–101 nrendo.2013.223 [pii]; 10.1038/nrendo.2013.223 [doi] 24247220

[23] Schietke R, Warnecke C, Wacker I, Schodel J, Mole DR, et al (2010) The lysyl oxidases LOX and LOXL2 are necessary and sufficient to repress E-cadherin in hypoxia: insights into cellular transformation processes mediated by HIF-1. J Biol Chem 285: 6658–6669 M109.042424 [pii]; 10.1074/jbc.M109.042424 [doi] 20026874 PMC2825461

[24] Hammond SM (2006) MicroRNAs as oncogenes. Curr Opin Genet Dev 16: 4–9. 16361094 10.1016/j.gde.2005.12.005

[25] Liu CY, Xu JY, Shi XY, Huang W, Ruan TY, et al (2013) M2-polarized tumor-associated macrophages promoted epithelial-mesenchymal transition in pancreatic cancer cells, partially through TLR4/IL-10 signaling pathway. Lab Invest 93: 844–854 labinvest201369 [pii]; 10.1038/labinvest.2013.69 [doi] 23752129

[26] Bonde AK, Tischler V, Kumar S, Soltermann A, Schwendener RA (2012) Intratumoral macrophages contribute to epithelial-mesenchymal transition in solid tumors. BMC Cancer 12: 35 1471-2407-12-35 [pii]; 10.1186/1471-2407-12-35 [doi] 22273460 PMC3314544

[27] Baay M, Brouwer A, Pauwels P, Peeters M, Lardon F (2011) Tumor cells and tumor-associated macrophages: secreted proteins as potential targets for therapy. Clin Dev Immunol 2011: 565187 10.1155/2011/565187 [doi] 22162712 PMC3227419

[28] Yang J, Weinberg RA (2008) Epithelial-mesenchymal transition: at the crossroads of development and tumor metastasis. Dev Cell 14: 818–829. 18539112 10.1016/j.devcel.2008.05.009

[29] Aigner A (2011) MicroRNAs (miRNAs) in cancer invasion and metastasis: therapeutic approaches based on metastasis-related miRNAs. J Mol Med 10.1007/s00109-010-0716-0 21234533

[30] Dykxhoorn DM, Wu Y, Xie H, Yu F, Lal A, et al (2009) miR-200 enhances mouse breast cancer cell colonization to form distant metastases. PLoS One 4: e7181. 19787069 10.1371/journal.pone.0007181 PMC2749331

[31] Gregory PA, Bert AG, Paterson EL, Barry SC, Tsykin A, et al (2008) The miR-200 family and miR-205 regulate epithelial to mesenchymal transition by targeting ZEB1 and SIP1. Nat Cell Biol 10: 593–601. 18376396 10.1038/ncb1722

[32] Sayed D, Abdellatif M (2011) MicroRNAs in development and disease. Physiol Rev 91: 827–887. 21742789 10.1152/physrev.00006.2010

[33] Varambally S, Cao Q, Mani RS, Shankar S, Wang X, et al (2008) Genomic loss of microRNA-101 leads to overexpression of histone methyltransferase EZH2 in cancer. Science 322: 1695–1699. 19008416 10.1126/science.1165395 PMC2684823

[34] Cao Q, Yu J, Dhanasekaran SM, Kim JH, Mani RS, et al (2008) Repression of E-cadherin by the polycomb group protein EZH2 in cancer. Oncogene 27: 7274–7284. 18806826 10.1038/onc.2008.333 PMC2690514

[35] Watanabe S, Ueda Y, Akaboshi S, Hino Y, Sekita Y, et al (2009) HMGA2 maintains oncogenic RAS-induced epithelial-mesenchymal transition in human pancreatic cancer cells. Am J Pathol 174: 854–868. 19179606 10.2353/ajpath.2009.080523 PMC2665746

[36] Guo L, Chen C, Shi M, Wang F, Chen X, et al (2013) Stat3-coordinated Lin-28-let-7-HMGA2 and miR-200-ZEB1 circuits initiate and maintain oncostatin M-driven epithelial-mesenchymal transition. Oncogene 32: 5272–5282 onc2012573 [pii]; 10.1038/onc.2012.573 [doi] 23318420

[37] Chen Z, Cheng Q, Ma Z, Xi H, Peng R, et al (2013) Overexpression of RKIP Inhibits Cell Invasion in Glioma Cell Lines through Upregulation of miR-98. Biomed Res Int 2013: 695179 10.1155/2013/695179 [doi] 24392454 PMC3874320

[38] Tavazoie SF, Alarcon C, Oskarsson T, Padua D, Wang Q, et al (2008) Endogenous human microRNAs that suppress breast cancer metastasis. Nature 451: 147–152 nature06487 [pii]; 10.1038/nature06487 [doi] 18185580 PMC2782491

[39] Png KJ, Yoshida M, Zhang XH, Shu W, Lee H, et al (2011) MicroRNA-335 inhibits tumor reinitiation and is silenced through genetic and epigenetic mechanisms in human breast cancer. Genes Dev 25: 226–231 25/3/226 [pii]; 10.1101/gad.1974211 [doi] 21289068 PMC3034897

[40] Mitchell PS, Parkin RK, Kroh EM, Fritz BR, Wyman SK, et al (2008) Circulating microRNAs as stable blood-based markers for cancer detection. Proc Natl Acad Sci U S A 105: 10513–10518. 18663219 10.1073/pnas.0804549105 PMC2492472

[41] Ryder M, Gild M, Hohl TM, Pamer E, Knauf J, et al (2013) Genetic and pharmacological targeting of CSF-1/CSF-1R inhibits tumor-associated macrophages and impairs BRAF-induced thyroid cancer progression. PLoS One 8: e54302 10.1371/journal.pone.0054302 [doi];PONE-D-12-29549 [pii] 23372702 PMC3553126

