# Supplementary material for: miRNA Expression in Anaplastic Thyroid Carcinomas
Source: PLoS One. 2014 Aug 25;9(8):e103871. doi: 10.1371/journal.pone.0103871 (PMC4143225; doi:10.1371/journal.pone.0103871)
Supplement: Figure S1 — Expression levels of 13 miRNA measured by qRT-PCR in 3 ATC studied by microarrays and in 3 ATC independant or obtained from the literature. The microarray expressions are included for comparision. Log2 ratios represent the expression ratios of the genes in the tumors versus the normal tissues. (PPT) [file pone.0103871.s001.ppt]

## Slide 1
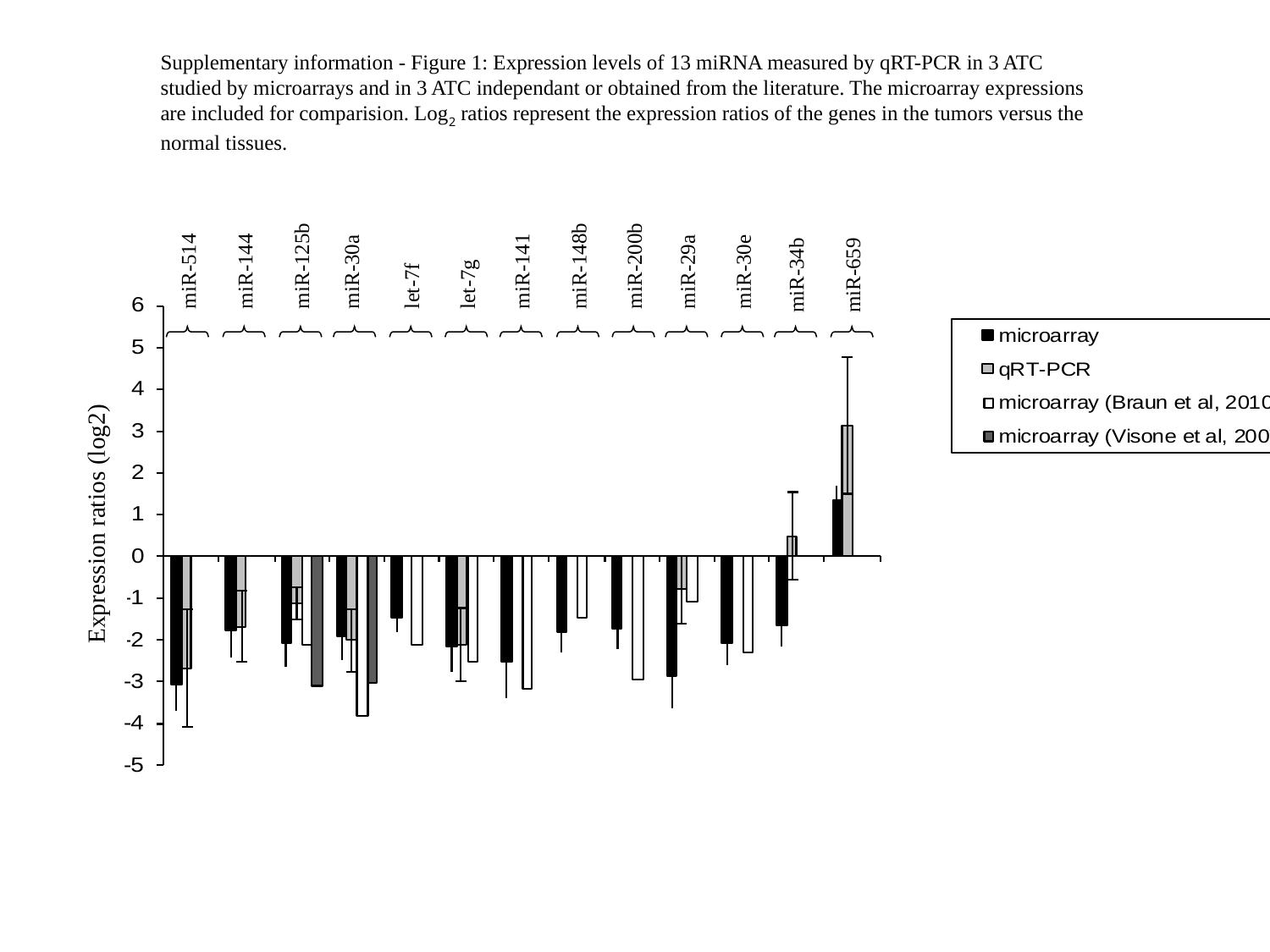

Supplementary information - Figure 1: Expression levels of 13 miRNA measured by qRT-PCR in 3 ATC studied by microarrays and in 3 ATC independant or obtained from the literature. The microarray expressions are included for comparision. Log2 ratios represent the expression ratios of the genes in the tumors versus the normal tissues.
miR-148b
miR-125b
miR-200b
miR-514
miR-144
miR-141
miR-30a
miR-29a
miR-30e
let-7g
let-7f
miR-34b
miR-659
Expression ratios (log2)
