# Supplementary material for: miRNA Expression in Anaplastic Thyroid Carcinomas
Source: PLoS One. 2014 Aug 25;9(8):e103871. doi: 10.1371/journal.pone.0103871 (PMC4143225; doi:10.1371/journal.pone.0103871)
Supplement: Table S4 — Deregulated miRNA in the 11 ATC samples and their corresponding values in papillary carcinoma (PC), follicular carcinoma (FC), follicular adenoma (FA) and autonomous adenoma (AA). MiRNA expression values are in bold if they varied from the baseline by at least 1.5-fold (log2 of expression ratios). (PPTX) [file pone.0103871.s005.pptx]

## Slide 1
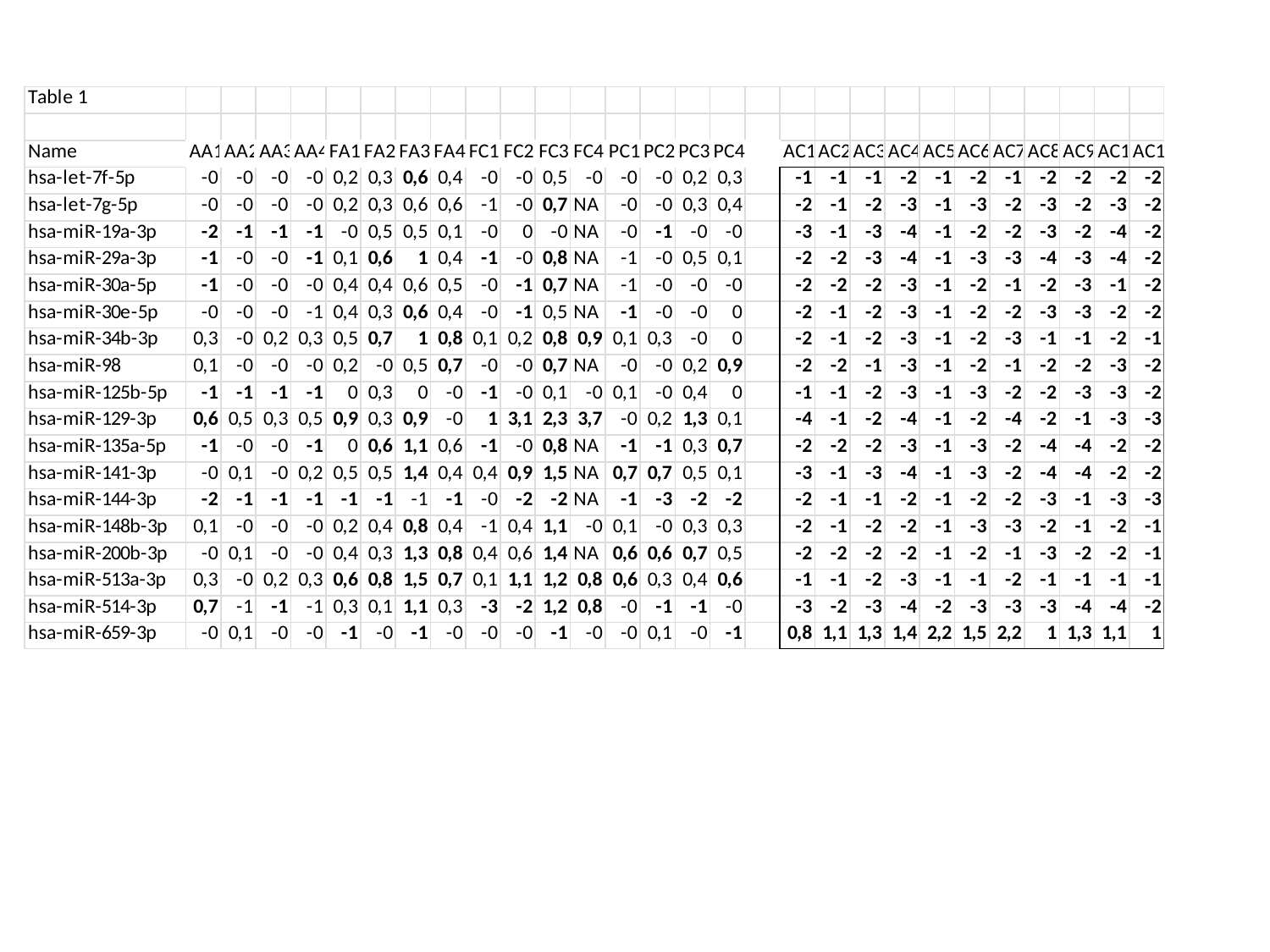

## Slide 2
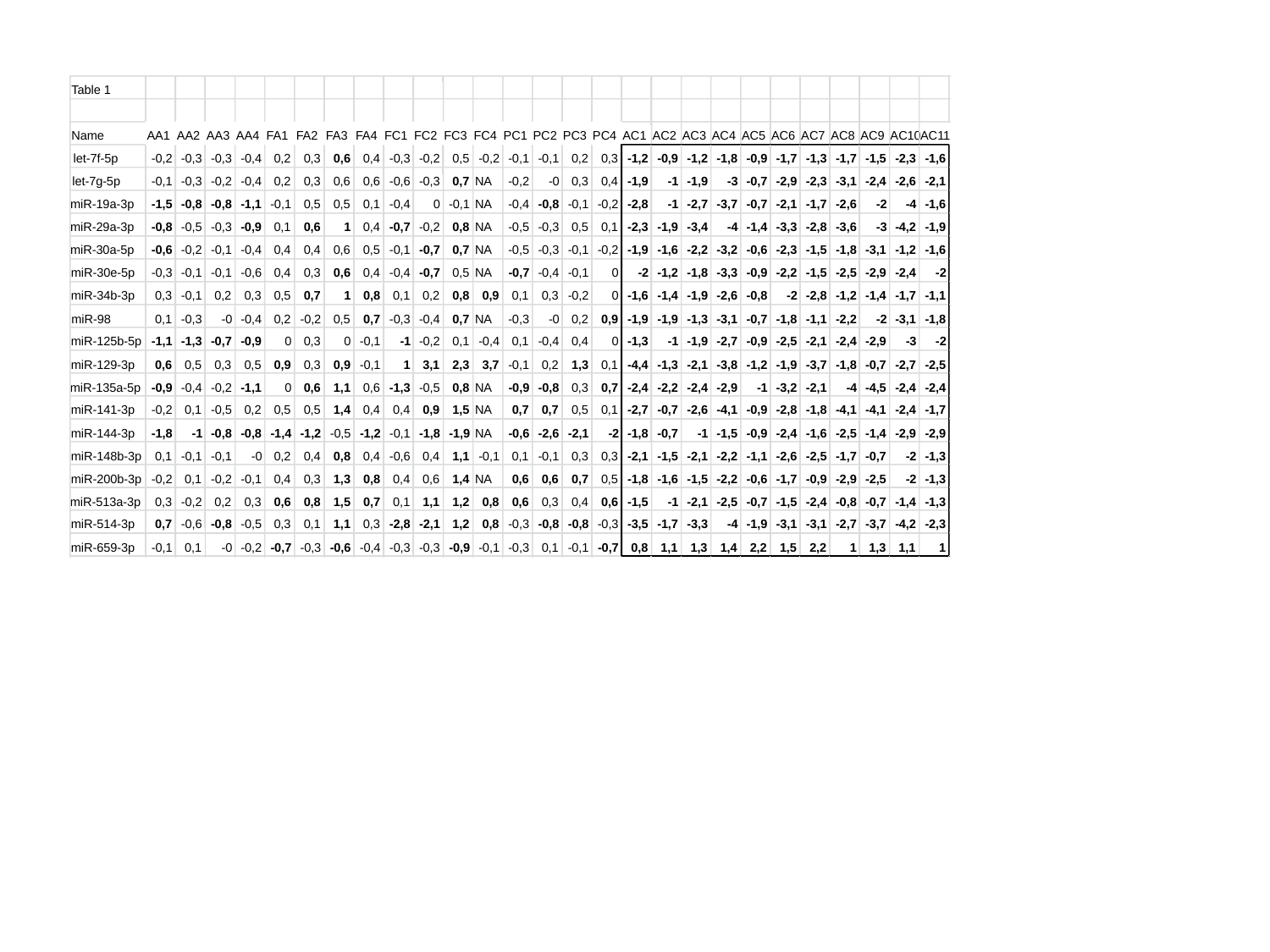

0,4
0,8
0,4
-0,6
0,4
1,1
-0,1
0,1
-0,1
0,3
0,3
-2,1
-1,5
-2,1
-2,2
-1,1
-2,6
-2,5
-1,7
-0,7
-2
-1,3
miR-200b-3p
-0,2
0,1
-0,2
-0,1
0,4
0,3
1,3
0,8
0,4
0,6
1,4
NA
0,6
0,6
0,7
0,5
-1,8
-1,6
-1,5
-2,2
-0,6
-1,7
-0,9
-2,9
-2,5
-2
-1,3
miR-513a-3p
0,3
-0,2
0,2
0,3
0,6
0,8
1,5
0,7
0,1
1,1
1,2
0,8
0,6
0,3
0,4
0,6
-1,5
-1
-2,1
-2,5
-0,7
-1,5
-2,4
-0,8
-0,7
-1,4
-1,3
miR-514-3p
0,7
-0,6
-0,8
-0,5
0,3
0,1
1,1
0,3
-2,8
-2,1
1,2
0,8
-0,3
-0,8
-0,8
-0,3
-3,5
-1,7
-3,3
-4
-1,9
-3,1
-3,1
-2,7
-3,7
-4,2
-2,3
miR-659-3p
-0,1
0,1
-0
-0,2
-0,7
-0,3
-0,6
-0,4
-0,3
-0,3
-0,9
-0,1
-0,3
0,1
-0,1
-0,7
0,8
1,1
1,3
1,4
2,2
1,5
2,2
1
1,3
1,1
1
Table 1
Name
AA1
AA2
AA3
AA4
FA1
FA2
FA3
FA4
FC1
FC2
FC3
FC4
PC1
PC2
PC3
PC4
AC1
AC2
AC3
AC4
AC5
AC6
AC7
AC8
AC9
AC10
AC11
let-7f-5p
-0,2
-0,3
-0,3
-0,4
0,2
0,3
0,6
0,4
-0,3
-0,2
0,5
-0,2
-0,1
-0,1
0,2
0,3
-1,2
-0,9
-1,2
-1,8
-0,9
-1,7
-1,3
-1,7
-1,5
-2,3
-1,6
let-7g-5p
-0,1
-0,3
-0,2
-0,4
0,2
0,3
0,6
0,6
-0,6
-0,3
0,7
NA
-0,2
-0
0,3
0,4
-1,9
-1
-1,9
-3
-0,7
-2,9
-2,3
-3,1
-2,4
-2,6
-2,1
miR-19a-3p
-1,5
-0,8
-0,8
-1,1
-0,1
0,5
0,5
0,1
-0,4
0
-0,1
NA
-0,4
-0,8
-0,1
-0,2
-2,8
-1
-2,7
-3,7
-0,7
-2,1
-1,7
-2,6
-2
-4
-1,6
miR-29a-3p
-0,8
-0,5
-0,3
-0,9
0,1
0,6
1
0,4
-0,7
-0,2
0,8
NA
-0,5
-0,3
0,5
0,1
-2,3
-1,9
-3,4
-4
-1,4
-3,3
-2,8
-3,6
-3
-4,2
-1,9
miR-30a-5p
-0,6
-0,2
-0,1
-0,4
0,4
0,4
0,6
0,5
-0,1
-0,7
0,7
NA
-0,5
-0,3
-0,1
-0,2
-1,9
-1,6
-2,2
-3,2
-0,6
-2,3
-1,5
-1,8
-3,1
-1,2
-1,6
miR-30e-5p
-0,3
-0,1
-0,1
-0,6
0,4
0,3
0,6
0,4
-0,4
-0,7
0,5
NA
-0,7
-0,4
-0,1
0
-2
-1,2
-1,8
-3,3
-0,9
-2,2
-1,5
-2,5
-2,9
-2,4
-2
miR-34b-3p
0,3
-0,1
0,2
0,3
0,5
0,7
1
0,8
0,1
0,2
0,8
0,9
0,1
0,3
-0,2
0
-1,6
-1,4
-1,9
-2,6
-0,8
-2
-2,8
-1,2
-1,4
-1,7
-1,1
miR-98
0,1
-0,3
-0
-0,4
0,2
-0,2
0,5
0,7
-0,3
-0,4
0,7
NA
-0,3
-0
0,2
0,9
-1,9
-1,9
-1,3
-3,1
-0,7
-1,8
-1,1
-2,2
-2
-3,1
-1,8
miR-125b-5p
-1,1
-1,3
-0,7
-0,9
0
0,3
0
-0,1
-1
-0,2
0,1
-0,4
0,1
-0,4
0,4
0
-1,3
-1
-1,9
-2,7
-0,9
-2,5
-2,1
-2,4
-2,9
-3
-2
miR-129-3p
0,6
0,5
0,3
0,5
0,9
0,3
0,9
-0,1
1
3,1
2,3
3,7
-0,1
0,2
1,3
0,1
-4,4
-1,3
-2,1
-3,8
-1,2
-1,9
-3,7
-1,8
-0,7
-2,7
-2,5
miR-135a-5p
-0,9
-0,4
-0,2
-1,1
0
0,6
1,1
0,6
-1,3
-0,5
0,8
NA
-0,9
-0,8
0,3
0,7
-2,4
-2,2
-2,4
-2,9
-1
-3,2
-2,1
-4
-4,5
-2,4
-2,4
miR-141-3p
-0,2
0,1
-0,5
0,2
0,5
0,5
1,4
0,4
0,4
0,9
1,5
NA
0,7
0,7
0,5
0,1
-2,7
-0,7
-2,6
-4,1
-0,9
-2,8
-1,8
-4,1
-4,1
-2,4
-1,7
miR-144-3p
-1,8
-1
-0,8
-0,8
-1,4
-1,2
-0,5
-1,2
-0,1
-1,8
-1,9
NA
-0,6
-2,6
-2,1
-2
-1,8
-0,7
-1
-1,5
-0,9
-2,4
-1,6
-2,5
-1,4
-2,9
-2,9
miR-148b-3p
0,1
-0,1
-0,1
-0
0,2
